# Enhancing medical staff participation in blood donation: Insights into willingness, motivations and policy expectations

**DOI:** 10.1097/MD.0000000000042489

**Published:** 2025-05-16

**Authors:** Jun Li, Xinyi Jiang

**Affiliations:** a Chongqing Blood Center, Chongqing, China.

**Keywords:** blood donation, policy expectations, promotional mechanisms, willingness

## Abstract

Medical staff hold dual roles as advocates and potential blood donors, yet systemic barriers within high-pressure clinical environments hinder their participation. Understanding their motivations and challenges is critical for strengthening blood supply systems. This cross-sectional study aimed to identify determinants of donation willingness and policy expectations among medical staff to inform evidence-based interventions. A structured questionnaire, developed via Delphi methodology with hematologists, policy experts, and psychologists, was administered to medical staff at a tertiary Grade A hospital in Chongqing, China. Stratified random sampling ensured representation across demographics. Data were collected via an encrypted online platform (SoJump.com) and analyzed using SPSS 22.0 and GraphPad Prism 6. Among 1096 participants, the observation group (non-donors, n = 460, 41.97%) exhibited statistically significant demographic divergences from the control group across gender, age, educational attainment and professional category (all *P* < .05). Multivariate logistic regression identified these variables as independent predictors of donation status (*P* < .05). Despite 83.26% of the observation group endorsing blood donation and 69.34% comprehending deferral criteria, key participation barriers included occupational workload saturation (59.13%), inadequate promotional mechanisms (49.57%), adverse physiological reactions (43.04%), and insufficient motivation (26.52%). For intervention optimization, respondents in the observation group prioritized structured promotional mechanisms (80.22%), establishing policy guidance protocols (53.26%), and systematizing regular donor recruitment activities (46.74%). Young, male, and highly educated medical staff exhibited higher donation rates. Structural reforms to alleviate occupational burdens, optimize donor recruitment paradigms, and institutionalize periodic mobilization are imperative to align clinical demand with donor supply.

## 1. Introduction

Blood donation is a profoundly meaningful social welfare activity that benefits personal health and plays a crucial role in saving lives.^[1]^ It reduces cardiovascular risks, enhances immunity, and fosters societal cohesion.^[2,3]^ Over the past 2 decades, China has transitioned to a voluntary non-remunerated donation system, achieving a donation rate of 12.2 per 1000 population by 2023. Standard whole-blood donations (200–400 mL/session) require a 6-month interval, while current promotional mechanisms encompass prioritized blood access for donors and families alongside honor certificates. Despite this progress, a persistent gap remains compared to high-income nations. Critically, the number of active repeat donors remain insufficient to meet growing clinical demands driven by aging populations and expanded healthcare access. This donor recruitment challenge persists globally.^[4,5]^ Blood donors remain a minority demographic even in developed countries.^[6]^ There are various barriers to donating blood that influence people behavior towards blood donation, such as cultural beliefs, lifestyle, fear of reduced health, and other issues.^[7–10]^

Within this context, medical staff occupy dual roles as advocates and potential donors. Despite their clinical familiarity with transfusion-related exigencies, systemic barriers such as occupational workload saturation and insufficient promotional mechanisms remain understudied within China high-pressure healthcare ecosystem. Prior research has predominantly focused on public donor motivations, overlooking the unique paradox faced by medical staff who champion donation campaigns yet encounter institutionalized barriers to personal participation. Therefore, this study investigated their donation willingness and policy expectations to address this paradox.

## 2. Materials and methods

### 2.1. Questionnaire design and validation

Given the absence of validated instruments assessing medical staff donation behaviors under regional policy frameworks, a structured questionnaire was developed via Delphi methodology with hematologists, policy experts, and psychologists. The finalized instrument comprised 3 modules: demographic characteristics, donation willingness, and policy expectations.

### 2.2. Sampling and data acquisition

Sample size estimation utilized Cochran formula for categorical variables, targeting a 95% confidence interval with a ± 3% margin of error, yielding a minimum requirement of 1067 participants. The study employed stratified random sampling at a tertiary Grade A comprehensive teaching hospital in Chongqing, China, a nationally leading healthcare institution that integrates clinical services, medical education, interdisciplinary research, preventive care, and international health programs. The response rate of the medical staff to the questionnaire was 100%. Group allocation was based on self-reported donation behavior in the questionnaire. The control group comprised medical staff with prior blood donation experience, whereas the observation group included those without any donation history. Data collection was conducted via SoJump.com, ensuring encrypted dissemination and real-time monitoring. This study was approved by the Chongqing Blood Center Ethics Committee. All methods strictly complied with the relevant guidelines and regulations.

### 2.3. Statistical analysis

Data were analyzed using SPSS 22.0 (IBM Corp., Armonk) and GraphPad Prism 6 (GraphPad Software, San Diego). Categorical variables were expressed as frequencies (%) and compared via *χ*² tests. Multivariate logistic regression models identified predictors of donation behavior, incorporating covariates such as gender, age, educational attainment, and professional category. Odds ratios and 95% confidence intervals were reported. Statistical significance was set at *α* = 0.05 (two-tailed).

### 2.4. Data availability

Data were anonymized prior to collection. Data sharing requires approval from the Ethics Committee.

## 3. Results

### 3.1. Donation experience and determinants

Among 1096 participants, the observation group (non-donors, n = 460, 41.97%) exhibited statistically significant demographic divergences from the control group across gender, age, educational attainment, and professional category (all *P* < .05, Table 1). Multivariate logistic regression identified these variables as independent predictors of donation status (*P* < .05, Table 2).

**Table 1:** The blood donation experience of the participants (n = 1096).

Characteristics		Control group (n = 636)	Observation group (n = 460)	*χ* ^2^	*P*
Gender	Male	357 (32.57%)	178 (16.24%)	32.48	.01
	Female	279 (25.46%)	282 (25.73%)		
Age	18–25	161 (14.69%)	213 (19.43%)	54.48	.01
	26–35	249 (22.72%)	139 (12.68%)		
	36–45	149 (13.59%)	64 (5.84%)		
	46–55	77 (7.03%)	44 (4.01%)		
Educational attainment	Doctoral	43 (3.92%)	6 (0.55%)	109.13	.01
	Master	130 (11.86%)	49 (4.47%)		
	Bachelor	331 (30.20%)	179 (16.33%)		
	Below bachelor	132 (12.04%)	226 (20.62%)		
Professional category	Doctors	142 (12.96%)	106 (9.67%)	39.21	.01
	Nurses	140 (12.77%)	102 (9.31%)		
	Laboratory technicians	125 (11.41%)	32 (2.92%)		
	Other	229 (20.89%)	220 (20.07%)		

**Table 2:** Multivariate logistic regression analysis of blood donation.

Variable	*β*	S.E.	Wald *χ*^2^	*P*	OR	95% CI
Gender	0.57	0.12	21.28	.00	1.77	[1.39, 2.24]
Age	−0.23	0.07	12.26	.00	0.79	[0.69, 0.90]
Educational attainment	0.46	0.09	26.32	.00	1.58	[1.33, 1.88]
Professional category	−0.35	0.10	12.45	.00	0.71	[0.58, 0.86]

Categorical variables were coded as follows: Gender (Male = 1, Female = 0); Age (18–25 = 1, 26–35 = 2, 36–45 = 3, 46–55 = 4); Educational Level (Doctoral = 1, Master = 2, Bachelor = 3, Below Bachelor = 4); Profession (Doctors = 1, Nurses = 2, Laboratory Technicians = 3, Other = 4).

CI = confidence intervals, OR = odds ratios.

### 3.2. Non-donor attitudes and barriers

**Figure 1.**
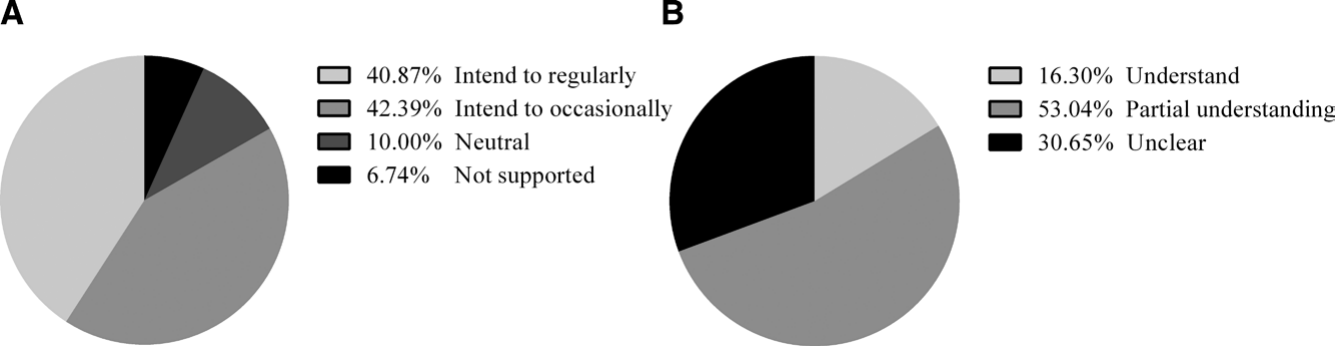
The willingness towards blood donation in the observation group (n = 460).

**Figure 2.**
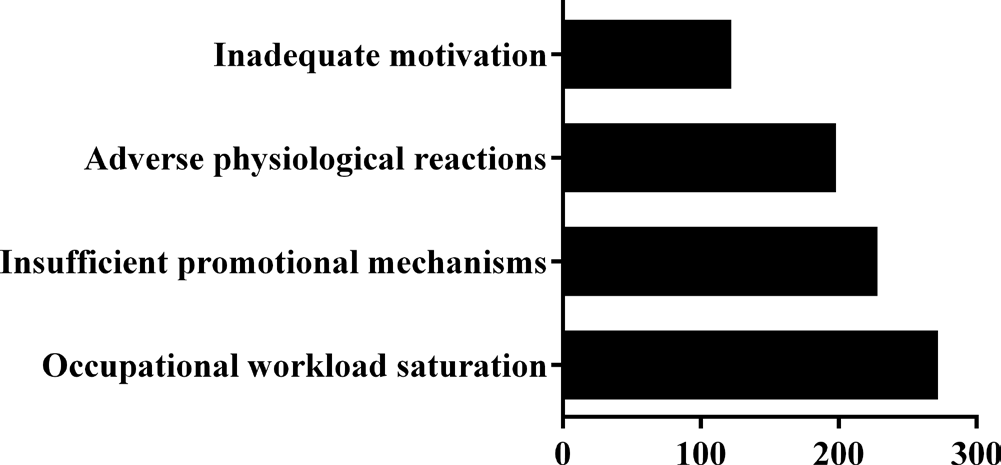
The reasons for nonparticipation in blood donation in the observation group (n = 460).

As shown in Figure 1, the majority (83.26%) of the observation group clearly expressed their support for blood donation, and 69.34% demonstrated a comprehensive understanding of unsuitable conditions for blood donation. Among them, 40.87% planned to donate blood regularly and 42.39% did so occasionally. A small percentage (10.00%) maintained a neutral stance on blood donation without any particular inclination or bias, whereas 6.74% stated that they would not participate due to perceived risks. However, they were unable to participate in blood donation for various reasons, as illustrated in Figure 2. These reasons included occupational workload saturation (59.13%), insufficient promotional mechanisms (49.57%), adverse physiological reactions (43.04%), and inadequate motivation (26.52%).

### 3.3. Intervention preferences

**Figure 3.**
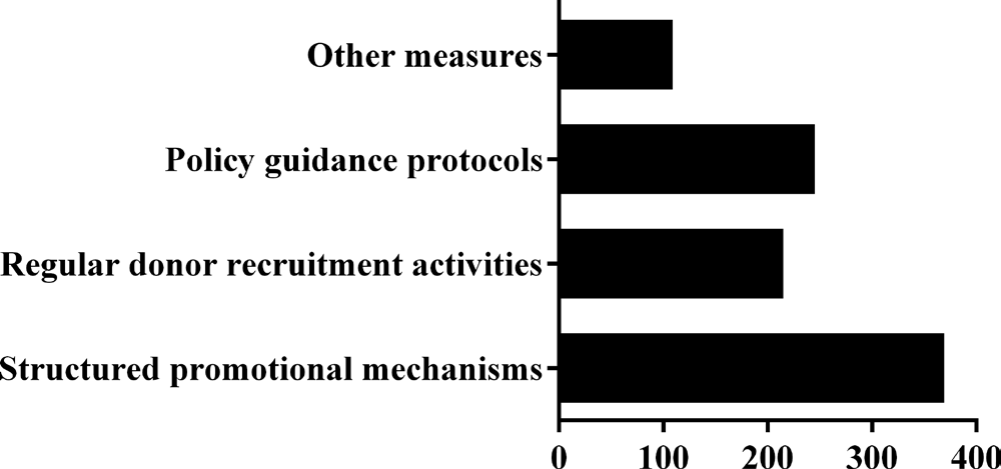
The observation group on blood donation expectations (n = 460).

As delineated in Figure 3, observation group respondents prioritized 3 intervention strategies to enhance donation engagement: implementing structured promotional mechanisms (80.22%), establishing policy guidance protocols (53.26%), and systematizing regular donor recruitment activities (46.74%).

## 4. Discussion

This study sheds light on the multiple barriers and drivers affecting medical staff participation in blood donation, which highlights their conflicting roles as both advocates and constrained donors.^[11]^ Based on these findings, we propose recommendations to address this paradox through targeted interventions.

### 4.1. Key barriers to participation

Based on the data, a total of 1096 medical staff participated in this survey. Among them, 636 (58.03%) reported previous blood donations, while 460 (41.97%) reported no blood donation experience. There were significant differences in gender, age, educational attainment and professional category, with young male medical staff of higher educational levels exhibiting greater donation likelihood. Similar to studies in Germany and Bangladesh,^[12,13]^ demographic skewness toward younger males exhibiting advanced educational attainment was observed within the cohort. This demographic pattern may stem from advanced medical literacy and heightened social responsibility associated with educational attainment, both of which potentially bolster confidence in navigating donation procedures. However, China unique occupational pressures contrast with high-income nations’ focus on donor convenience and public awareness campaigns. Among the observation group, most medical staff clearly expressed their support for blood donation and were knowledgeable about which conditions were unsuitable for blood donation. Only 6.74% of the medical staff held differing opinions, suggesting that a minority may have reservations about the potential risks of blood donation.

Blood donation is a pivotal activity within social welfare endeavors with significant implications for patient treatment and life-saving.^[14]^ The World blood donor day is observed on June 14 every year with the aim of encouraging more individuals to voluntarily donate blood and advocate for the implementation of the global blood safety program. As key participants and advocates of blood donation, medical staff play a crucial role in promoting the advancement of this undertaking.^[15]^ Occupational workload saturation emerged as the predominant barrier to donation engagement to blood donation among the medical staff in the observation group, accounting for approximately 59.13%. Additionally, insufficient promotional mechanisms and adverse physiological reactions were significant issues, accounting for 49.57% and 43.04% respectively. The issue of inadequate motive was found to be relatively mild, accounting for 26.52% of the total. This aligns with similar concerns raised by other blood donors.^[16–19]^

### 4.2. Strategic interventions for enhanced engagement

An overwhelming 80.22% of the observation group supported refining promotional strategies through behavioral economics principles, with initiatives like continuing medical education credits and flexible post-donation recuperative leave receiving particular endorsement. However, ethical considerations surrounding incentivization warrant caution, as excessive reliance on material rewards may inadvertently commodify altruistic acts.^[20,21]^ Furthermore, 53.26% of the observation group acknowledged the critical role of effective policy guidance in promoting blood donation. Using posters, flyers, social media campaigns, and word-of-mouth referrals can contribute to enhancing awareness and motivation among medical staff to donate blood.^[22]^ Disseminating tailored awareness campaigns targeting this specific group can encourage more active participation in blood donation initiatives. Notably, nearly half of the medical staff (46.74%) indicated that organizing regular blood donation activities would effectively motivate them to participate in donation. This suggests that such activities create a convenient platform for medical staff to contribute to overcoming any apprehensions or fears associated with blood donation. Interestingly, a minority (23.70%) of the surveyed medical staff suggested that implementing additional measures could enhance blood donation promotion efforts. The measures include providing comprehensive services and support at the blood donor center, offering flexible donation schedules, and strengthening blood management and utilization practices.^[23,24]^ Addressing these issues comprehensively may give the medical staff greater confidence, leading to increased participation in blood donation.

### 4.3. Demographic-specific insights and innovations

Medical staff who are young, male, and highly educated demonstrate elevated donation rates, a phenomenon linked to disparities in health literacy. Those with advanced education typically exhibit stronger comprehension of uniform donation procedures and qualification standards. However, occupational health burdens such as circadian rhythm disruption caused by rotational shifts, prolonged pathogen exposure, and increased hypertension rates lead to temporary donor disqualifications. Despite constituting a significant portion of the workforce, female medical staff exhibited lower participation rates, a disparity that may stem from physiological constraints or socioprofessional pressures. Demographically tailored interventions, such as iron supplementation programs, may ameliorate these disparities. Similarly, younger professionals may benefit from mentorship programs pairing novice donors with experienced colleagues to alleviate procedural anxieties.

This study contributes novel insights by delineating the interplay between occupational workload saturation and donation behavior within China high-pressure healthcare ecosystem, advancing the discourse on donor recruitment strategies tailored to high-stress professional environments. Unlike prior research focused on general populations, our findings specifically highlight the unique challenges faced by medical staff, emphasizing how systemic pressures and workload intensity intersect with their capacity to engage in donation-related activities. These results deepen the understanding of context-specific barriers and opportunities for optimizing donor participation in demanding occupational settings.

### 4.4. Limitations and future directions

This study has several limitations. The cross-sectional design restricts causal inferences and precluded assessment of donation frequency among existing donors. Self-reported data might be influenced by social desirability bias, potentially inflating donation willingness estimates. Geographically, the sample was limited to tertiary hospitals in Chongqing, necessitating future multi-center studies across diverse regions to improve generalizability. Longitudinal research should be prioritized to investigate donation frequency dynamics, health status impacts on donor eligibility, and behavioral patterns among regular donors. These extensions could yield deeper insights into donation mechanisms and inform targeted strategies for sustaining donor engagement.

## 5. Conclusions

Young, male, and highly educated medical staff exhibited higher donation rates. Structural reforms to alleviate occupational burdens, optimize donor recruitment paradigms, and institutionalize periodic mobilization are imperative to align clinical demand with donor supply.

## Author contributions

**Formal analysis:** Jun Li.

**Data curation:** Xinyi Jiang.

**Investigation:** Xinyi Jiang.

**Writing – original draft:** Jun Li.

**Writing – review & editing:** Xinyi Jiang.

## References

[1] The Lancet Haematology. Celebrating blood donors amid advances in blood transfusion. Lancet Haematol. 2023;10:e389. 37263713 10.1016/S2352-3026(23)00129-1

[2] Peffer K den Heijer M de Kort WLAM Verbeek ALM Atsma F. Cardiovascular risk in 159 934 frequent blood donors while addressing the healthy donor effect. Heart. 2019;105:1260–5. 30872386 10.1136/heartjnl-2018-314138

[3] Bani-Ahmad MA Khabour OF Gharibeh MY Alshlool KN. The impact of multiple blood donations on the risk of cardiovascular diseases: insight of lipid profile. Transfus Clin Biol. 2017;24:410–6. 28797569 10.1016/j.tracli.2017.07.001

[4] Al-Riyami AZ Burnouf T Wood EM.; ISBT COVID-19 Convalescent Plasma Working Group. International society of blood transfusion survey of experiences of blood banks and transfusion services during the COVID-19 pandemic. Vox Sang. 2022;117:822–30. 35262978 10.1111/vox.13256 PMC9115426

[5] Ferguson E Edwards ARA Masser BM. Simple reciprocal fairness message to enhance non-donor’s willingness to donate blood. Ann Behav Med. 2022;56:89–99. 34050653 10.1093/abm/kaab026

[6] Kassie A Azale T Nigusie A. Intention to donate blood and its predictors among adults of Gondar city: using theory of planned behavior. PLoS One. 2020;15:e0228929. 32119662 10.1371/journal.pone.0228929 PMC7051045

[7] Hyde MK Masser BM Coundouris SP. A review of whole-blood donors’ willingness, motives, barriers and interventions related to donating another substance of human origin. Transfus Med. 2022;32:95–114. 35068004 10.1111/tme.12849

[8] Gilchrist PT Masser BM Horsley K Ditto B. Predicting blood donation intention: the importance of fear. Transfusion. 2019;59:3666–73. 31663615 10.1111/trf.15554

[9] Keten HS Isik O Kus C. Determination of the knowledge level, attitudes, and behaviors of islamic religious officials toward blood donation. Transfus Apher Sci. 2017;56:875–9. 29133024 10.1016/j.transci.2017.10.006

[10] Chaurasia R Patidar GK Pandey HC. Critical appraisal of knowledge, attitude and practice studies for blood donation in India. Transfus Med. 2023;33:197–204. 36941796 10.1111/tme.12968

[11] Khatun R Otaibi BW Ssentongo A Hazelton JP Cooper AB. Medical student attitudes toward blood donation in times of increased need. Am Surg. 2022;88:2338–44. 33877939 10.1177/00031348211011083

[12] Stock B Mockel L. Characterization of blood donors and non-blood donors in Germany using an online survey. Health Technol (Berl). 2021;11:595–602. 33680702 10.1007/s12553-021-00532-y PMC7920751

[13] Quader MA. Socio-demographic characteristics of blood donor in a tertiary care specialized hospital. Bangladesh J Med. 2021;32:113–9.

[14] Brunson DC Belanger GA Sussmann H Fine AM Pandey S Pham TD. Factors associated with first-time and repeat blood donation: adverse reactions and effects on donor behavior. Transfusion. 2022;62:1269–79. 35510783 10.1111/trf.16893

[15] Hughes JD Hernandez MC Jenkins DH. Survey of medical center employees’ willingness and availability to donate blood in support of a civilian warm fresh whole blood program. Am J Disaster Med. 2019;14:101–11. 31637691 10.5055/ajdm.2019.0321

[16] Prakash S Das PK Mishra D. Incidence and risk predictors analysis of adverse donor reactions in whole blood donation. Transfus Clin Biol. 2020;27:207–12. 33027707 10.1016/j.tracli.2020.09.003

[17] Bharadwaj L Shetty A Chowdappa V. Knowledge, inhibition and incentives towards voluntary blood donation: a comparative study among medical and non-medical students. Natl J Lab Med. 2019;8:5–7.

[18] Thijsen A Gemelli CN Davison TE Masser B. Using the health action process approach to predict blood donation intentions and return behavior following a vasovagal reaction for whole blood and plasma donors. Transfusion. 2022;62:1791–8. 35924722 10.1111/trf.17052

[19] Yang J Fan D Xie D. First donor haemovigilance system at a national level in China: establishment and improvement. Vox Sang. 2023;118:357–66. 36896482 10.1111/vox.13421

[20] Irving AH Harris A Petrie D. A systematic review and network meta-analysis of incentive- and non-incentive-based interventions for increasing blood donations. Vox Sang. 2020;115:275–87. 32043603 10.1111/vox.12881

[21] Zhu T Yeo NTB Gao SY Loke GG. Inventory-responsive donor-management policy: a tandem queueing network model. Manuf Serv Oper Manag. 2023;25:1585–602.

[22] Saha S Chandra B. A cross-sectional blood study in India: from donation activities of donors to blood bank services. Current Sci. 2016;110:1789–800.

[23] Martin-Santana JD Melian-Alzola L. The influence of service quality and anticipated emotions on donor loyalty: an empirical analysis in blood centres in Spain. Health Care Manag Sci. 2022;25:623–48. 35841450 10.1007/s10729-022-09600-9 PMC9674737

[24] Melián-Alzola L Martín-Santana JD. Service quality in blood donation: satisfaction, trust and loyalty. Serv Bus. 2020;14:101–29.

